# Material strategy for strain-invariant radio-frequency electronics

**DOI:** 10.1088/2631-7990/ae569e

**Published:** 2026-04-08

**Authors:** Senhao Zhang, Furong Yang, Chaoyun Song, Huanyu Cheng

**Affiliations:** 1 Department of Engineering Science and Mechanics, The Pennsylvania State University, University Park, PA 16802, United States of America; 2 Department of Engineering, King’s College London, London WC2R 2LS, United Kingdom

Wearable and stretchable biointegrated electronics rely on integrated radio-frequency (RF) modules for control, power transfer, and communication, yet skin deformation disrupts resonance and degrades impedance matching and efficiency. The resonant frequency is determined by the effective electrical length and permittivity, but only the electrical length responds to tensile strain, leading to strain-sensitive behavior. A recent study reports a novel dielectric elastomer, termed a dielectro-elastic elastomer (DEE) with mechanically tunable permittivity for suppressing frequency shifts in RF electronics^[^1^]^. This tunable strategy enables strain-invariant RF performance and a skin-interfaced wireless health monitor with a stable 30 m operating range under 30% strain.

## 1. Principle of tunable permittivity in DEE composites

**Figure 1.**
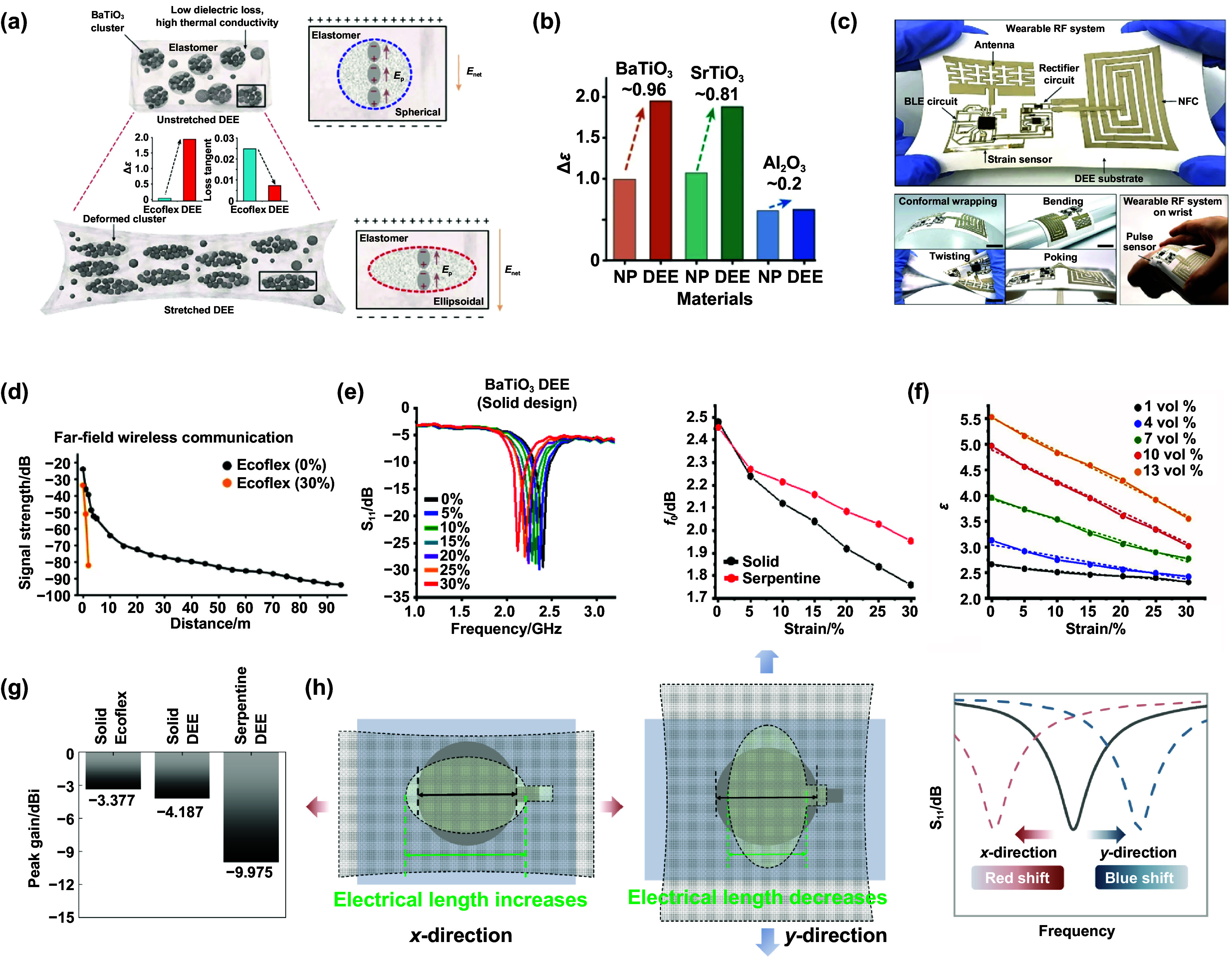
Material design of dielectro-elastic elastomers (DEE) for strain-invariant radio-frequency electronics. (a) Illustration of the DEE composite substrate comprising BaTiO_3_ clusters. (b) Comparison of permittivity change under 30% strain between uniformly mixed composites and DEE composites incorporating high-*κ* nanoparticles (BaTiO_3_, SrTiO_3_, and Al_2_O_3_). (c) Photograph of a strain-invariant stretchable wireless system. (d) Measured receiving signal strength by the stretchable Ecoflex-based antenna before and after 30% stretching, with a transmitter positioned at different distances. (e) *S*-parameter (*S*_11_) curves of the serpentine stretchable patch antenna fabricated on DEE. And comparison of resonance change of the solid-patch and serpentine-patch antennas on conventional elastomer under applied strain from 0 to 30%. (f) Graph showing the decrease in permittivity under an applied strain at different volume fractions. (g) Simulated peak gain comparison of the Ecoflex-/DEE-based solid patch antenna and DEE-based serpentine patch antenna (with parameters taken from the reported design in the ref.1). (h) Schematics illustrating direction-dependent strain effects on electrical length (left and middle) and resonance behavior (right). (a)–(f) Reproduced from^[^1^]^, with permission from Springer Nature.

Dielectric elastomers typically consist of a soft elastomer matrix with embedded particles that deform from spherical to ellipsoidal under stretching, enabling strain-tunable permittivity. Inherently deformable particles, such as liquid metal, enable the shape reconfiguration required for tunable permittivity, but their low-*κ* and higher loss compromise RF performance. By contrast, conventional high-*κ* ceramic fillers (e.g., TiO_2_, BaTiO_3_, PbTiO_3_) exhibit limited deformability at the particle level within elastomer composites^[^2^]^. To overcome the trade-off between deformability and dielectric performance, a clustering strategy encapsulates BaTiO_3_ nanoparticles into deformable microscale spheres, allowing the resulting clusters, rather than isolated particles, to reconfigure their shapes under strain (Figure 1(a)). Specifically, DEE is synthesized by dissolving Ecoflex in dichloromethane with dispersed BaTiO_3_ nanoparticles, followed by water addition that destabilizes the interfaces and rapidly induces polarity-driven clustering. Compared with the permittivity changes (Δ*ϵ*) of ∼1.0 from the BaTiO_3_-particle-mixing strategy, the clustering strategy exhibits a larger Δ*ϵ* of ∼2.10 and ∼1.95 at 13.56 MHz and 2.4 GHz (30% strain) by enhanced depolarization enabled from cluster deformation, thereby satisfying the permittivity requirement of stretchable RF electronics (Figure 1(b)). Meanwhile, the DEE exhibits a lower loss tangent value of 0.0074 at 2.4 GHz than that of 0.026 from Ecoflex, owing to the intrinsically low loss of the BaTiO_3_ particles. This generic clustering strategy can be extended to diverse high-*κ* materials, broadening the functionality of stretchable RF electronics. Overall, the clustering-enabled DEE bridges a critical materials gap, offering a manufacturable, tunable dielectric platform that simultaneously supports efficient strain-induced permittivity change and low RF losses for strain-insensitive stretchable RF electronics.

## 2. Strain-invariant stretchable RF electronics

The mechanically tunable permittivity of the DEE translates directly into strain-invariant RF performance at the device and system levels. Specifically, this stands in sharp contrast to stretchable patch antennas built on conventional elastomers, where even small strains (<5%**–**10%) trigger 20.8% redshifts in resonance, severely degrading the far-field wireless link. In addition, DEE-based inductive coils (13.56 MHz) preserve resonance and efficiency under 30% strain, and coplanar transmission lines maintain S_11_/S_21_ from 50 kHz to 6 GHz without band shift or insertion loss. Building on the improvements of the above devices, a skin-interfaced wireless health monitor with mechanical and electromagnetic stability is demonstrated, which is crucial for wearable applications (Figure 1(c)). Overall, the clustering-enabled DEE provides a novel paradigm for stretchable RF electronics with material-level electro-mechanical coupling to preserve wireless performance. This shift enables robust long-range communication, efficient power transfer, and stable signal integrity in dynamic, skin-interfaced environments, laying the foundation for the next-generation wireless wearables.

## 3. Challenges, limitations, and prospects

As a minor issue that requires correction, the communication performance of the Ecoflex-based antenna under 30% stretching appears to degrade much more rapidly with distance than that of the unstrained case (Figure 1(d)). In theory, the two curves should be roughly parallel under identical measurement conditions according to the Friis equation.

Despite the remarkable progress enabled by clustering-based DEEs, several intrinsic limitations remain. The DEE lacks sufficient dielectric variation to counteract the resonance change of traditional patch antennas (Figure 1(e)), and further increasing the BaTiO_3_ volume fraction beyond 10 vol% yields no further Δ*ϵ* increase (Figure 1(f)). As a result, a serpentine layout has been adopted, but this design, together with the high permittivity of DEE (despite its low loss), inherently drives the antenna to an electrically small geometry (∼*λ*_0_/10), causing significant degradation in both peak gain and radiation efficiency (Figure 1(g)). The achieved resonant-frequency stability at the cost of degraded radiation efficiency is unacceptable for long-range communication. In comparison, even a detuned traditional patch antenna can potentially offer higher radiation efficiency than the reported electrically small antenna. Thus, it is imperative to develop strain-insensitive traditional antennas for practical use in wearable scenarios. Looking forward, integrating clustering-based DEEs with reconfigurable RF layouts, metasurface-inspired structures, and/or hybrid liquid-metal/ceramic fillers may further expand the design space.

Moreover, conventional planar RF devices such as traditional patch antennas, waveguides, and transmission lines inherently show anisotropic electromagnetic responses under directional stretching. In particular, when the tensile strain is applied perpendicular to the current path, the contraction due to Poisson’s effect shortens the electrical length and induces a blue shift^[^3^]^, which cannot be compensated by the DEE because its permittivity can only decrease (Figure 1(h)). Therefore, for conventional patch antennas, the isotropic permittivity response of the DEE can, at best, be tuned to compensate deformation along a single principal strain direction. For other strain directions, the anisotropic electromagnetic behavior of these devices leads to a residual frequency shift. For future multi-directional strain-insensitive RF devices without compromising radiation performance, it would be highly desirable to develop dielectric elastomers to realize sufficient anisotropic permittivity variation. Prospectively, delicate control over the initial filler morphology or the inter-particle/cluster distance can introduce distinct permittivity-strain behaviors^[^4^]^ to open up a new pathway toward engineering anisotropic DEE materials. In summary, the clustering-enabled DEE provides a compelling pathway toward strain-invariant RF electronics and thus warrants further investigation and optimization in future research.
